# The Use of Bioelectrical Impedance Analysis Measures for Predicting Clinical Outcomes in Critically Ill Children

**DOI:** 10.3389/fnut.2022.847480

**Published:** 2022-06-06

**Authors:** Zi-Hong Xiong, Xue-Mei Zheng, Guo-Ying Zhang, Meng-Jun Wu, Yi Qu

**Affiliations:** ^1^Department of Pediatric Intensive Care Unit, Chengdu Women’s and Children’s Central Hospital, University of Electronic Science and Technology of China, Chengdu, China; ^2^Key Laboratory of Birth Defects and Related Diseases of Women and Children (Ministry of Education), Department of Pediatrics, West China Second University Hospital, Sichuan University, Chengdu, China; ^3^University of Electronic Science and Technology of China, Chengdu, China; ^4^Department of Anesthesiology, Chengdu Women’s and Children’s Central Hospital, University of Electronic Science and Technology of China, Chengdu, China

**Keywords:** phase angle, critical illness, children, bioimpedance analysis, progonosis

## Abstract

**Background:**

The study aimed to investigate the association of bioelectrical impedance analysis (BIA) for predicting clinical outcomes in critically ill children.

**Methods:**

This single-center prospective observational study included patients admitted to a mixed Pediatric Intensive Care Unit (PICU). All patients underwent anthropometric measurement and BIA measurements in the first 24 h of admission. The patients were classified into different groups based on body mass index (BMI) for age. Electronic hospital medical records were reviewed to collect clinical data for each patient. All the obtained data were analyzed by the statistical methods.

**Results:**

There were 231 patients enrolled in our study, of which 31.6% were diagnosed with malnutrition. The phase angle (PhA) of 90-day survivors was significantly higher than that of the non-survivors (4.3° ± 1.1°vs. 3.1° ± 0.9°, *P* = 0.02). The age-adjusted Spearman partial correlation analysis showed a weak negative correlation between PhA and duration of medical ventilation (r_s_ = -0.42, *P* < 0.05). Furthermore, length of stay in PICU has a very weak correlation with ECW/TBW (r_s_ = 0.29, *P* < 0.05), and a negative correlation with protein (r_s_ = -0.27, *P* < 0.05). Multivariate analysis found that PhA was a significant predictor associated with the 90-day mortality when it was adjusted for PRISM III score (adjusted OR = 1.51, CI: 1.10–2.07, *p* = 0.01). The area under the ROC (AUROC) of PhA for predicting 90-day mortality was 0.69 (95% CI: 0.53–0.85, *p* < 0.05), and the cutoff value of PhA was 3.0°, with a sensitivity and specificity of 83 and 53%, respectively.

**Conclusion:**

BIA-derived PhA was found to be an independent predictor of 90-day mortality among critically ill children. A low PhA was associated with a prolonged duration of medical ventilation.

## Introduction

Malnutrition is highly prevalent in critically ill childrenin the pediatric intensive care unit (PICU) (1). Critically ill children usually experience total body water redistribution, edema, systemic metabolic status changes, and rapid lean body mass loss, thereby leading to their body composition changes dramatically and nutritional status deterioration. Worse nutritional status is often associated with unfavorable clinical consequences such as prolonged duration of mechanical ventilation, longer ICU (intensive care unit) length of stay, extended hospitalization length, and increased hospital mortality (2). However, the most used methods to assess the nutritional state including weight loss, body mass index (BMI), and biochemical indicators do not accurately reflect the real alteration of the nutritional status among critically ill children.

Bioelectrical impedance analysis (BIA) is a method of assessing body composition and its use in ICU patients has progressively increased (3). BIA measures impedance by applying a current through the body. The impedance consists of two components: resistance (R) and reactance (Xc). Human tissues have different contents of fluid and conducting electrolytes, thus the resistance of tissues is different. Human cell membranes and tissue interfaces can affect capacitive reactance. Therefore, body composition can be estimated by using BIA. Several studies have confirmed that BIA was a useful tool for assessing body composition and has a good relative agreement with dual-energy X-ray absorptiometry (DXA) in children (4, 5). Moreover, BIA is a portable, easy-to-use, non-invasive, and low-cost method. For this reason, coupled with children’s comfort and cooperation, BIA is significantly appropriate for children in daily clinical practice.

Recently, some studies have pointed out that BIA can be served as a good predictor to evaluate the disease severity among critically ill adults (6), especially BIA-derived phase angle (PhA). PhA was calculated using the following equation: PhA = (Xc/R)×(180/π). It can reflect the integrity of the cellular membrane, total body cell mass, and hydration status of the body (4, 5). Lower PhA always represent the poorer the prognosis. A systematic review found PhA seemed to be a good indicator of mortality in many clinical situations (7). However, studies on assessing the association between BIA measurements parameters and clinical outcomes in PICU patients are lacking.

This present study aimed to determine whether BIA measurements parameters on PICU admission independently predicts adverse clinical outcomes among critically ill children.

## Materials and Methods

### Study Design and Setting

We conducted a prospective observational single-center study at the PICU of a tertiary hospital from March 2019 to November 2021. Our PICU is a mixed ICU that receives medical and surgical patients. This study was approved by the Chengdu Women’s and Children’s Central Hospital in Sichuan, China [Registration No. 2019(11)].

Inclusion criteria were patients admitted to the PICU with an age range of 1–18 years. The exclusion criteria were as follows: (1) patients with any amputation, or with skin injury on the area where the electrodes of the BIA instrument would be placed, (2) underwent dialysis 2 h before BIA, (3) with congenital chromosomal abnormalities or hereditary metabolic diseases, (4) with serious errors in their BIA results. Written informed consent was obtained from parents and/or legal guardians.

### Anthropometric Measurement and Classification of Nutritional Status

Anthropometric measurement was performed in the first 24 h of admission. To reduce the possibility of errors, all measurements were performed by a trained PICU physician. Weight was measured using a scale that was calibrated for accuracy before each use. Infants were weighed using a scale accurate to 5 g. Children who could not be weighed standing were held by an adult. The child’s weight was obtained by subtracting the weight of the adult from the total weight of the child and adult. Children aged 3 years or younger assumed the supine position for the measurement. Length was measured using a pediatric anthropometer with an accuracy of 0.1 cm. In children aged older than 3 years, height was measured using a stadimeter with an accuracy of 0.1 cm. In children whose condition prevented the use of conventional measuring techniques (e.g., patients who were mechanically ventilated or taking vasoactive drugs and above 1 m), the ulna length was measured using a pediatric anthropometer. Height and length prediction was extrapolated by Gauld et al. (8). Arm muscle circumference (AMC) was measured using a metric tape marked in 0.5-cm increments. Measurements were taken at the midpoint of the distance between the acromion and olecranon with the arm extended along the body.

BMI was calculated using the following equation: BMI/A = W (kg)/H^2^ (m). Nutritional status was classified based on BMI for age using the World Health Organization (WHO) growth charts as the reference. Patients were categorized as the non-malnourished group, moderately malnourished group, and severely malnourished group, defined by weight for age 0 to –2 standard deviation (SD),–2 to –3 *SD*, and less than –3 *SD* of the WHO growth charts, respectively.

### Demographic, Clinical, and Biochemical Data

Hospital electronic medical records (EMR) were reviewed to collect data for each patient, including age, diagnosis, height, weight, length of hospital stay, length of PICU stay, duration of mechanical ventilation (MV), and other notable characteristics. Hospital Mortality was defined as death during PICU stay. The patients who died at 90 days after PICU admission were ascertained by hospital EMR review, telephone contact, and clinical follow-up data. Blood was collected within 24 h after admission. Blood biochemical indexes, such as serum albumin level, total lymphocyte count (TLC), and hemoglobin level were also collected from EMR. Pediatric Risk of Mortality (PRISM) score which is based on 14 routinely measured, physiologic variables, and 23 variable ranges, was collected at the time of admission as a prognostic indicator, and judging severity of the disease.

### Bioelectrical Impedance Analysis Data

InBodyS10 (Biospace, Seoul, South Korea) was used for the measurements of the study. The device uses an alternating current of 220 volts with a frequency of 50 kHz. Patients were immobilized in a supine position with their arms were separated from the trunk and feet comfortably apart. Subsequently, surface electrodes were placed on the patient’s thumbs and middle fingers and two sides of the ankles, after disinfection with 70% alcohol. The results of the resistance (R) and reactance (Xc) were obtained after sending a weak electric current through the body. BIA measurements were directly displayed on the device, including intracellular water, extracellular water (ECW) and total body water (TBW), ECM/TBW, body cell mass, bone mineral content, skeletal muscle mass, FM (fat mass), % body fat (%BF), TBW/FFM (fat-free mass), proteins, and minerals. All participants underwent BIA within 24 h after admission.

### Statistical Analysis

Statistical analysis was performed using the SPSS 20.0 (IBM Corp, Armonk, NY, United States). Normalities of data distributions were confirmed using the Kolmogorov—Smirnov test. Normally distributed quantitative data are presented as the mean ± standard deviation (Mean ± *SD*). Categorical variables were presented as frequencies and proportions. Abnormally distributed continuous variables are presented as the median (25th and 75th percentiles). Continuous variables were compared and analyzed based on the independent sample *t*-test and analysis of variance. Qualitative data were compared by X^2^ test. Age-adjusted Spearman partial correlation analysis was performed to explore the correlation between the BIA measurements and duration of MV, length of ICU stay, and length of hospital. Univariate and multivariable logistic regression analyses were performed to determine the predictive factors of 90-day mortality. Odds ratios (ORs) with 95% confidence intervals (CIs) were calculated. Receiver operating characteristic (ROC) curves were established to evaluate the predictive abilities value of phase angle. The area under the ROC curve (AUC) and its 95% confidence interval (CI) were measured. The optimal cutoff values for ROC curves were established using the Youden index. *P*-value < 0.05 was considered statistically significant.

## Results

A total of 231 patients were included during the study period. All patients were divided into three groups according to their BMI. Among them, 158 were non-malnourished, 34 were moderately malnourished, and 39 were severely malnourished. There were significant differences in ages, duration of mechanical ventilation, and 90-day mortality among the three groups (*P* < 0.05). In the severely malnourished group, the longest duration of MV and the highest mortality were seen. No statistically significant difference was found in length or height, weight, PRISM III score, the percentage of invasive mechanical ventilation, mortality in PICU, length of stay in PICU, and length of stay in hospital in different nutritional status groups. The comparison of general characteristics and clinical outcomes of patients is described in Table 1.

**TABLE 1:** Comparison of general characteristics, clinical outcomes among the different nutrition status groups (*n* = 231).

Variables	Non- malnourished (*n* = 158)	Moderately malnourish (*n* = 34)	Severely malnourished (*n* = 39)	*P*-value
Age (years)	1.90 (1.10–4.38)	2.40 (1.53–5.30)	4.90 (1.70–10.00)	0.03*
Male sex (%)	73.50%	71.40%	63.20%	0.68
Length or height (cm)	87.50 (78.00–107.00)	94.50 (78.25–114.25)	115.00 (82.00–132.00)	0.14
Weight (Kg)	12.50 (10.30–16.00)	11.25 (8.88–16.50)	14.00 (8.50–22.50)	0.58
PRISM III score	8.48 ± 3.94	10.32 ± 4.64	11.69 ± 3.71	0.48
MV, *n* (%)	56 (35.4)	10 (29.4)	19 (48.7)	0.50
Duration of MV, days	5.00 (3.00–7.00)	5.00 (4.00–14.00)	11.50 (6.00–16.00)	0.02*
PICU mortality, *n* (%)	5 (2.16%)	2 (0.086%)	4 (1.73%)	0.48
90-day mortality	6 (2.59%)	3 (1.30%)	15 (6.4%)	0.02*
LOS in PICU, days	7.00 (6.00–10.00)	8.00 (3.00–11.75)	11.20 (2.75–15.00)	0.86
LOS in hospital, days	13.00 (9.00–27.75)	14.00 (10.50–22.00)	14.00 (10.00–22.00)	0.89

*P < 0.05.

*Quantitative data was shown as (x̄ ± SD) or median (25th and 75th percentiles). Qualitative data was showed as numbers (percentage). PRISM, pediatric risk of mortality; LOS, length of stay; MV, mechanical ventilation; PICU, pediatric intensive care unit.*

Table 2 provides a comparison of the nutritional indicators and BIA measurements between 90-day survivors and non-survivors. The results showed that the BMI, albumin levels, and PhA were significantly different between the two groups (*P* < 0.05). The PhA of 90-day survivors was significantly higher than that of the non-survivors (4.3° ± 1.1°vs. 3.1° ± 0.9°, *P* = 0.02). No statistical difference was observed in weight, AMC, TLC, hemoglobin, and the remaining BIA measurements.

**TABLE 2:** Comparison of the nutritional indicators and BIA measurements between 90-day survivors and non-survivors.

Variables	Survivors(*n* = 158)	Non-Survivors(*n* = 34)	*P*-value
Weight (Kg)	14.5 (10.0–16.0)	16.4 (12.1–21.5)	0.41
BMI, kg/m^2^	15.6 ± 2.4	13.9 ± 3.2	0.03*
AMC, cm	15.0 ± 1.3	15.5 ± 1.9	0.16
Albumin, g/dL	38.7 ± 6.3	37.2 ± 9.0	0.03*
TLC, cell/mm^3^	2.4 (1.3–3.1)	2.2 (0.9–4.3)	0.39
Hemoglobin, g/dL	109.8 (99.0–122.0)	120.5 (92.0–127.5)	0.24
PhA	4.3 ± 1.1	3.1 ± 0.9	0.02*
BCM, kg	7.6 (4.8–9.7)	8.3 (5.3–9.6)	0.81
BMC, kg	0.7 (0.3–1.0)	0.7 (0.5–1.0)	0.22
SMM, kg	4.9 (2.4–6.8)	5.4 (2.8–6.7)	0.74
FM, kg	2.2 (1.6–3.8)	2.3 (0.6–2.8)	0.45
%BF	18.3 (7.4–27.6)	17.7 (3.0–22.5)	0.87
Protein, kg	2.3 (1.5–2.9)	2.5 (1.6–2.9)	0.64
Mineral, kg	0.8 (0.4–1.2)	0.9 (0.6–1.1)	0.27
ICW	5.3 (3.4–6.8)	5.8 (3.7–6.7)	0.65
ECW	3.6 (2.4–4.3)	4.07 (2.62–4.37)	0.601
TBW	8.9 (5.67–11.1)	9.9 (6.3–11.9)	0.44
ECW/TBW	0.4 (0.3–0.4)	0.4 (0.3–0.4)	0.27
%TBW/FFM	74.7 ± 1.6	74.4 ± 1.5	0.55

*P < 0.05.

*Values shown are mean ± SD (standard deviation), number (percentage) or median [IQR]. BMI, body mass index; AMC, arm muscle circumference; TLC, total lymphocyte count; BCM, Body cell mass; BMC, bone mineral content; SMM, skeletal muscle mass; FM, fat mass; BF, body fat; ICW, intracellular water; ECW, extracellular water; TBW, total body water; TBW/FFM, total body water/fat free mass.*

Due to the significant differences in child’s age among the three nutrition status groups, we used the age-adjusted Spearman partial correlation analysis to explore the association between the BIA measurements and different clinical outcomes (Table 3). There was a weak negative correlation between PhA and the duration of medical ventilation (r_s_ = –0.42, *P* < 0.05). Furthermore, length of stay in PICU has a weak correlation with ECW/TBW (r_s_ = 0.29, *P* < 0.05), and a weak negative correlation with protein (r_s_ = –0.27, *P* < 0.05).

**TABLE 3:** Spearman’s correlation coefficients between BIA measurements and clinical outcomes among all admitted patients.

Variables	Duration of MV	LOS in PICU	LOS in hospital
PhA	−0.42*	–0.14	–0.15
BCM, kg	–0.03	–0.05	0.08
SMM	–0.01	–0.12	0.14
FM	0.07	–0.11	0.10
%BF	0.15	–0.05	–0.05
Protein, kg	–0.07	−0.27*	0.14
Mineral, kg	–0.02	–0.07	0.19
ICW	–0.13	–0.17	0.13
ECW	–0.15	0.15	0.17
TBW	–0.14	0.19	0.15
ECW/TBW	0.04	0.29*	0.14

*P < 0.05.

*BCM, body cell mass; BMC, bone mineral content; SMM, skeletal muscle mass; FM, fat mass; BF, Body fat; ICW, intracellular water; ECW, extracellular water; TBW, total body water; TBW/FFM, total body water/fat free mass; MV, medical ventilation; LOS, length of stay; PICU, pediatric intensive care unit.*

Variables with statistically significant differences (*p* < 0.05) between 90-day survivors and non-survivors groups were tested in univariate and multivariate logistic analysis with adjustment confounding factors (age and PRISM III score). Univariate logistic regression analysis showed that PhA (as a continuous variable) and PRISM III score were associated with 90-day mortality (PhA: odds ratio (OR) = 1.54, confidence interval (CI): 1.09–2.17, *p* = 0.01; PRISM III score: OR = 0.89, CI: 0.82–0.98, *p* = 0.04). BMI, albumin, and age were not associated with 90-day mortality, nor were they confounders for the effect of PhA on 90-day mortality. Multivariate analysis suggested that PhA was a significant predictor associated with the 90-day mortality when PhA was adjusted for PRISM III score (adjusted OR = 1.51, CI: 1.10–2.07, *p* = 0.01 PRISM III score also was an independent predictor (OR = 0.89, CI: 0.83–0.98, *p* = 0.01) (see Table 4). No significant differences were found for BMI and albumin.

**TABLE 4:** Logistic regression multivariate analysis of determinants of 90-mortality.

Variable	Univariable		Multivariable	
	Unadjusted OR (95% CI)	*P*-value	Adjusted OR (95% CI)	*P*-value
Phase angle (°)	1.54 (1.09, 2.17)	0.01*	1.51 (1.10, 2.07)	0.01*
PRISM III score	0.89 (0.82, 0.98)	0.04*	0.89 (0.83, 0.98)	0.01*
Age	1.12 (0.95, 1.31)	0.97		
BMI	0.98 (0.75, 1.26)	0.91		
Alb	0.97 (0.89, 1.06)	0.48		

*P < 0.05. PRISM: pediatric risk of mortality, BMI, body mass index; Alb, albumin. Age, gender, BMI, and PRISM III score-predicted mortality were tested for confounding.

*Age, gender, and BMI were no confounders. PRISM III score predicted mortality was a confounder and was adjusted for in the multivariable logistic regression analysis.*

**FIGURE 1:**
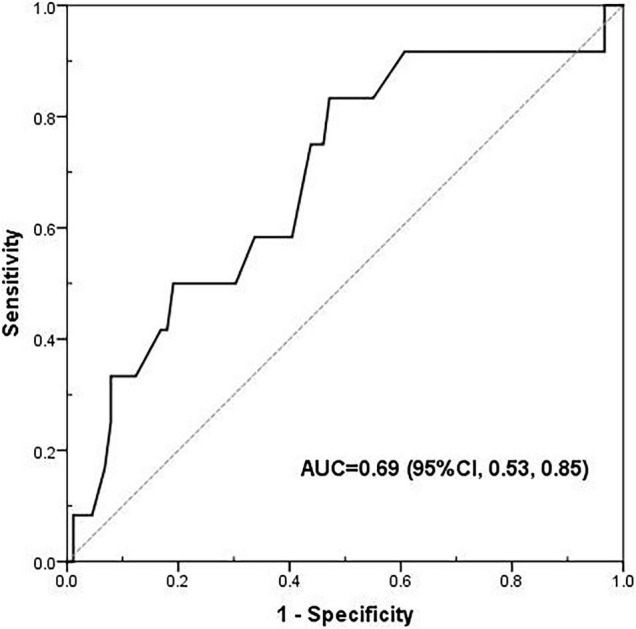
The receiver operating characteristic (ROC) curve of phase angle for 90-day mortality. The area under the ROC (AUROC) was 0.69 (95% CI: 0.53–0.85, *p* < 0.05), and the cut-off value of phase angle was 3.00°, with a sensitivity and specificity of 83 and 53%, respectively.

The results of the Receiver Operating Characteristic (ROC) curve analysis are provided in Figure 1. The area under the ROC (AUROC) of PhA was 0.69 (95% CI: 0.53–0.85, *p* < 0.05), and the cutoff value of PhA was 3.0°, with a sensitivity and specificity of 83 and 53%, respectively.

## Discussion

The development of both convenient and accurate methods for assessing the mortality or adverse clinical outcomes of PICU patients has been urgently needed, especially among children. Early identification and proper assessment of nutritional status are crucial to improve the patients’ outcomes (9–11). Bioelectrical impedance analysis (BIA) is a simple non-invasive assessment tool for body composition and therefore nutritional status. Beyond these, BIA is a rapid, low-cost, non-invasive, easy-to-perform, repeatable, and bedside feasible technique, it may be an alternative tool to death risk predictive scoring system to assess the severity of illness and predict the risk of mortality. This prospective observational study used BIA measurement parameters to assess the adverse clinical outcome of diseases in critically ill children. The result shows that the BIA-derived phase angle at PICU admission is an independent predictor of 90-day mortality. Children with PhA below 3° had 1.51 times higher adjusted risk of dying. A lower PhA was associated with a longer duration of mechanical ventilation (r_s_ = –0.42). Other BIA measurement parameters such as ECW/TBW and protein, had a very weak correlation with length of stay in PICU.

Malnutrition is common in critically ill children, with the prevalence rate of 31.6% (73/231) in our study. In contrast to adults, children are at a higher risk of experiencing malnutrition due to less nutritional stores and more nutrient consumption. Moreover, depleted muscle mass is associated with infectious complications, prolonged duration of MV, longer hospitalization, greater need for rehabilitation care after hospital discharge, and higher mortality (12). As this study showed, the patients in the malnourished group had a longer duration of MV and increased 90-day mortality than the normal nutritional status group (*P* < 0.05). Traditional anthropometric measurements might not accurately reflect body composition changes in life-threatening disease states. It is currently not possible to distinguish between overall weight loss or decreased BMI, this depletion comes from adipose tissue or muscle tissue. The multivariate regression analysis did not show an association between BMI and 90-day mortality, but rather between PhA and mortality in our study. Some studies showed that BIA measurements are better than anthropometry and blood biochemical analysis in the nutritional assessment of patients (4, 13–15). These findings emphasize the importance of body composition analysis to anthropometry in the ability of the nutrition assessment and predicting clinical outcomes.

BIA has unique advantages in this respect. Studies and systematic literature reviews (16, 17) have confirmed that BIA is a useful and reliable tool in the assessment of body composition in children and showed high specificity at detecting low muscle mass in patients. A recent study by Looijaard et al. (18) found that BIA-identified low skeletal muscle mass was consistent with CT scan, a golden standard method. Significant correlations have been detected for different BIA-derived muscle mass equations and CT-derived measurements (correlations ranging between 0.64 and 0.834). However, BIA also has some limitations. BIA measurements may have underestimated the presence of low muscle mass due to abnormal fluid redistribution in critically ill patients (19). Among younger infants (especially those aged less than 6 months), BIA may provide little benefit over anthropometry-based prediction equations. According to the result of previous studies, ECW/TBW ratios are useful and convenient tools used to assess the volume status of patients. the cut-off value for the assessment of edema was about 0.39 in adults (20–23). In our study, we measured the ECW/TBW ratio in all patients, and the median ECW/TBW ratio was greater than or equal to 0.40 in survivor and non-survivor groups. However, most of our patients did not display any edema symptoms. We hypothesize that children having higher water content than adults may be associated with a higher ECW/TCW ratio. There was a very weak association between length of stay in PICU and ECW/TCW ratio. It should be noted that BIA has intrinsic limitations in its ability to accurately distinguish between intravascular and interstitial volume in the extracellular compartment. Therefore, some recent studies have shown that bioelectrical impedance vector analysis, which visualizes impedance measurements (resistance and reactance), could be superior to any other parameter of the BIA for evaluating the hydration of critically ill patients in the ICU (24, 25).

BIA-derived PhA has been reported to be a good predictor of morbidity and mortality in different clinical situations (6, 26–28). PhA reflects the integrity of cell membranes and hydration status and is influenced by acute illness and general health. A low PhA always indicates cell membrane breakdown and decreased ability to store energy and complete metabolic functions. Considering the close correlation between PhA and nutrition status, it has been used to identify the patients at risk of nutrition status deterioration and worsening death. Stapel’s (29) study found that a PhA < 4.8° at ICU admission was an independent predictor of 90-day mortality in adults (adjusted odds ratio = 3.65, confidence interval: 1.34–9.93, *P* = 0.011). Zamberlan et al. (30). reported that among the BIA values, patients with PhA > 2.8°compared with patients with PhA ≤ 2.8° (*P* < 0.0001) showed a higher survival rate, and children with lower PhA values were more likely to remain in the PICU. Consistent with the result, we found that survivors showed significantly higher PhA compared with non-survivors. PhA < 3.0° at PICU admission was an independent predictor of 90-day mortality. In addition, we observed that lower PhA had a weak degree of correlation with the longer duration of MV. Other body composition parameters such as ECW, ICW, TBW, BCM, and skeletal muscle mass were not found to be an independent risk for death in our study. In our present study, significant differences in the levels of albumin and PRISM-III score were observed among the survivor group and the non-survivor group. Leite et al.’s (31) study showed that hypoalbuminemia at PICU admission is associated with higher 60-day mortality. However, we were not able to confirm the association between albumin and mortality. Several studies and meta-analyses (32–34) showed PRISM-III had good performance for mortality prediction in PICU. In our study, we confirmed that PRISM-III can be served as the indicator to an independently predictor of mortality.

Notably, PhA is affected by many factors, such as age, sex, level of physical activity, fluid status, and body composition (35). These factors contributed to the difficulty in analyzing the results among children. This is also the main limitation of the study. Furthermore, this was only a small single-center study and the results may not be generalizable. We still want to evaluate the clinical role of BIA measurements in critically ill children and establish appropriate PhA cut-points based on age, BMI, sex, and ethnicity in larger study populations.

## Conclusion

This study found that BIA-derived PhA can be considered an independent predictor of 90-day mortality in critically ill children. The not high specificity of PhA suggests that clinicians should have a comprehensive evaluation of the PhA in conjunction with the patient’s underlying disease status and other nutritional indicators, thereby contributing to clinical nutritional management and prognostic evaluation.

## Data Availability Statement

The raw data supporting the conclusions of this article will be made available by the authors, without undue reservation.

## Ethics Statement

The studies involving human participants were reviewed and approved by the Chengdu Women’s and Children’s Central Hospital, School of Medicine, UESTC. Written informed consent to participate in this study was provided by the participants’ legal guardian/next of kin.

## Author Contributions

G-YZ: conception and design of the research. Z-HX and G-YZ: the acquisition of the data and the analysis of the data. Z-HX and X-MZ: writing—original draft. Z-HX, YQ, and M-JW: writing—review and editing. All authors contributed to the article and approved the submitted version.

## Conflict of Interest

The authors declare that the research was conducted in the absence of any commercial or financial relationships that could be construed as a potential conflict of interest.

## Publisher’s Note

All claims expressed in this article are solely those of the authors and do not necessarily represent those of their affiliated organizations, or those of the publisher, the editors and the reviewers. Any product that may be evaluated in this article, or claim that may be made by its manufacturer, is not guaranteed or endorsed by the publisher.

## Abbreviations

AMC: Arm muscle circumference
BIA: Bioelectrical impedance analysis
BF: Body fat
BMI: Body mass index
BCM: Body cell mass
BMC: Bone mineral content
ECW: Extracellular water
FM: Fat mass
BF: body fat
FM: Fat mass
FFM: Fat-free mass
ICW: Intracellular water
LOS: Length of stay
MV: Mechanical ventilation
PICU: Pediatric intensive care unit
PhA: Phase angle
PICS: Pediatric Critical Illness Score
PRISM: Pediatric Risk of Mortality
SMM: Skeletal muscle mass
TLC: Total lymphocyte count
TBW: Total body water
TBW/FFM: Total body water/fat free mass
WHO: World Health Organization.
